# Brazilin Ameliorates High Glucose-Induced Vascular Inflammation via Inhibiting ROS and CAMs Production in Human Umbilical Vein Endothelial Cells

**DOI:** 10.1155/2014/403703

**Published:** 2014-03-02

**Authors:** Thanasekaran Jayakumar, Chao-Chien Chang, Shoei-Loong Lin, Yung-Kai Huang, Chien-Ming Hu, Antoinet Ramola Elizebeth, Shih-Chang Lin, Cheuk-sing Choy

**Affiliations:** ^1^Department of Pharmacology, College of Medicine, Taipei Medical University, Taipei, Taiwan; ^2^Department of Cardiology, Cathay General Hospital, Taipei, Taiwan; ^3^Department of Surgery, Taipei Hospital, Ministry of Health and Welfare, New Taipei City, Taiwan; ^4^School of Oral Hygiene, College of Oral Medicine, Taipei Medical University, Taipei, Taiwan; ^5^Taipei Medical University, Taiwan; ^6^Department of Biotechnology, Periyar Maniammai University, Thanjavur, Tamil Nadu, India; ^7^Division of Allergy and Immunology, Department of Internal Medicine, Cathay General Hospital, No. 280, Section 4, Jen-Ai Road, Taipei, Taiwan; ^8^The Laboratory of Allergy and Immunology, Cathay Medical Research Institute, New Taipei City, Taiwan; ^9^Department of Medicine, School of Medicine, Fu Jen Catholic University, New Taipei City, Taiwan; ^10^Emergency Department, Taipei Hospital, Ministry of Health and Welfare, No. 127, Su-Yuan Road, Hsin-Chuang, New Taipei City, Taiwan; ^11^Department of General Medicine, School of Medicine, College of Medicine, Taipei Medical University, Taipei, Taiwan

## Abstract

Vascular inflammatory process has been suggested to play a key role in the initiation and progression of atherosclerosis, a major complication of diabetes mellitus. Recent studies have shown that brazilin exhibits antihepatotoxic, antiplatelet, cancer preventive, or anti-inflammatory properties. Thus, we investigated whether brazilin suppresses vascular inflammatory process induced by high glucose (HG) in cultured human umbilical vein endothelial cells (HUVEC). HG induced nitrite production, lipid peroxidation, and intracellular reactive oxygen species formation in HUVEC cells, which was reversed by brazilin. Western blot analysis revealed that brazilin markedly inhibited HG-induced phosphorylation of endothelial nitric oxide synthase. Besides, we investigated the effects of brazilin on the MAPK signal transduction pathway because MAPK families are associated with vascular inflammation under stress. Brazilin blocked HG-induced phosphorylation of extracellular signal-regulated kinase and transcription factor NF-**κ**B. Furthermore, brazilin concentration-dependently attenuated cell adhesion molecules (ICAM-1 and VCAM-1) expression induced by various concentrations of HG in HUVEC. Taken together, the present data suggested that brazilin could suppress high glucose-induced vascular inflammatory process, which may be closely related with the inhibition of oxidative stress, CAMs expression, and NF-**κ**B activation in HUVEC. Our findings may highlight a new therapeutic intervention for the prevention of vascular diseases.

## 1. Introduction

Endothelial dysfunction is considered the primary cause in the pathogenesis of vascular disease in diabetes mellitus [1]. Diabetes is a metabolic disorder characterized by hyperglycemia and glucose intolerance due to lessened effectiveness of insulin action, insulin deficiency, or both. An increase of cardiovascular diseases due in part to hyperglycemia is associated with diabetes, which can induce endothelial dysfunction [2]. Alterations in endothelial function lead the development of insulin resistance [3]. The most likely cause of endothelial damage induced by hyperglycemia is the overproduction of reactive oxygen species (ROS) in mitochondria [4]. In aortic endothelial cells, hyperglycemia induces increases of mitochondrial superoxide production and prevents activity and expression of endothelial nitric oxide synthase (eNOS) [5]. ROS can modify endothelial function by a variety of mechanisms, such as peroxidation of membrane lipids, activation of NF-*κ*B, and decrease of the availability of nitric oxide (NO) [6]. Vascular disorders through overexpression of adhesion molecules are thought to play a role in the pathogenesis of atherosclerosis. Activation of NF-*κ*B induces adhesion molecules, such as VCAM-1 and ICAM-1, and, subsequently, induces an increase in the migration and adhesion of monocytic cells to endothelial cells, which are very important events during the inflammatory process.

Oxidative stress, due to the high glucose concentration, plays a vital role in the progress of diabetic impediments [7]. Previous studies have shown that high glucose activates nuclear factor-*κ*B (NF-*κ*B), one of the transcription factors for proinflammatory genes. NF-*κ*B is present in the cytoplasm as an inactive form bound to its inhibitor molecule, inhibitory factor of NF-*κ*B-*α* (I*κ*B-*α*). Translocation of NF-*κ*B from the cytoplasm to the nucleus is preceded by the phosphorylation, ubiquitination, and proteolytic degradation of I*κ*B-*α* [8]. It is assumed that high glucose-induced CAMs expression may depend on activation of NF-*κ*B. Under oxidative stress, endothelial cells generate ROS, such as superoxides and peroxynitrite, leading to low-density lipo protein (LDL) oxidation. The formation of ROS together with inflammatory factors including chemokines, cytokines, and adhesion molecules has been shown to be increased in atherosclerotic lesions [9]. Inflammatory responses, including inflammatory gene transcription, appear to involve free radicals or oxidative stresses, and thus free radical scavengers can suppress inflammatory gene expression. However, the effect of brazilin on high glucose-induced oxidative stress was not cleared in vascular endothelial cells.

Sappan Lignum, the heart wood of *Caesalpinia sappan* L., (*C. sappan* L) is used traditionally for large number of ailments and reported to have a wide variety of medicinal properties. The anti-inflammatory, antiproliferative, and antioxidant activities of *C. sappan* have been well documented [10, 11]. Brazilin [7,11b-dihydrobenz[b]indeno[1,2-d]pyran-3,6a,9,10(6H)-tetrol], the major component of *C. sappan* L. [12], is a natural red pigment, largely used for histological staining. Brazilin is also a promising chemopreventive agent as it is generally nontoxic and interferes with the process of carcinogenesis. Several synthetic types' brazilin analogues have demonstrated cancer-preventive properties towards a number of human cancer cell lines including HT29, A549, HL60, and K562 in MTT assays [13]. Brazilin induced vasorelaxation is reported to be inhibited by NG-nitro-Larginine methyl ester (L-NAME) and it is suggested that the mechanism by which brazilin caused vasodilation might be endothelial dependent [14]. This study was designed to investigate the effect and molecular mechanisms of brazilin on high-glucose stimulated human endothelial cells and the subsequent expression of CAMs in these cells. Our findings indicate a novel molecular mechanism underlying the therapeutic effects of brazilin for the prevention of vascular diseases.

## 2. Materials and Methods

### 2.1. Materials

**Figure 1:**
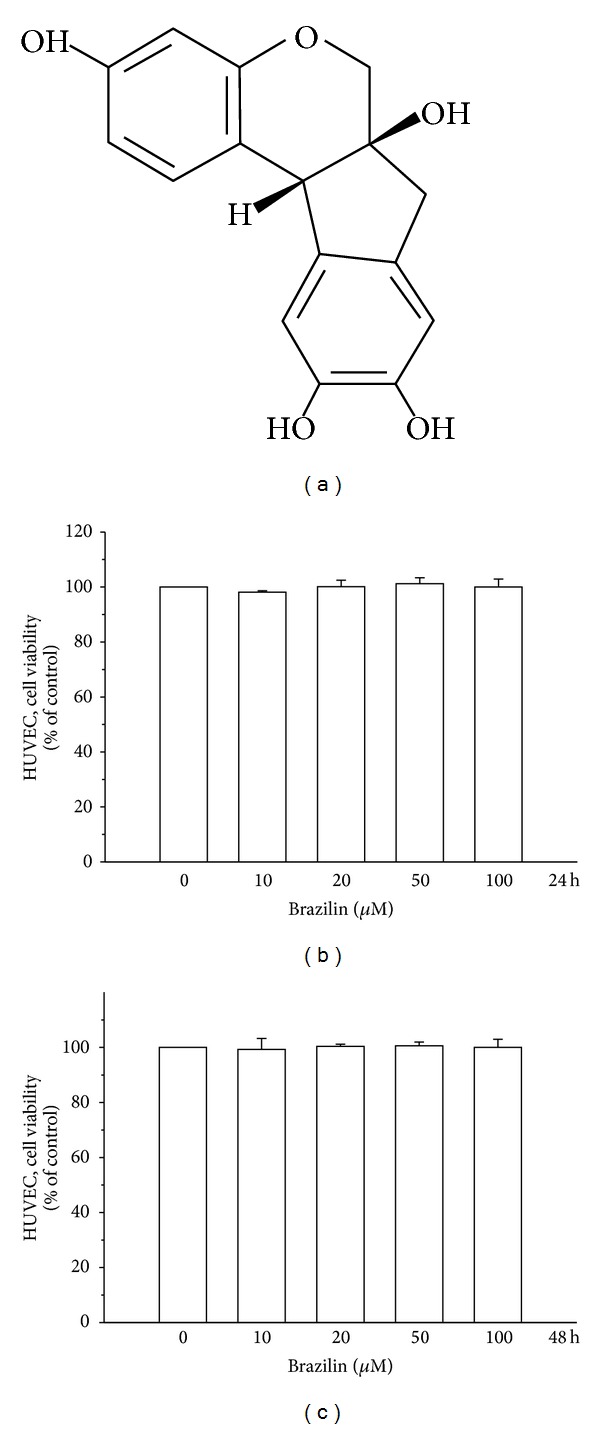
Effects of brazilin on the cell viability of human umbilical vein endothelial cells (HUVEC): (a) the structure of brazilin; (b) the viability of HUVECs during treatment with various concentrations (0–100 *μ*M) of brazilin for 24 h; (c) the viability of HUVECs during treatment with various concentrations (0–100 *μ*M) of brazilin for 48 h.

Brazilin (Figure 1(a)) was purchased from ICN Pharmaceuticals (Irvine, CA, USA). Cell culture reagents including M-199 medium, L-glutamine, penicillin, streptomycin, and fetal bovine serum were obtained from Gibco BRL (Grand Island, NY, USA). Antimouse and antirabbit immunoglobulin G-conjugated horseradish peroxidase (HRP) were purchased from Amersham Biosciences (Sunnyvale, CA, USA) and/or Jackson-Immuno Research (West Grove, PA, USA). A rabbit polyclonal antibody specific for NF-kB was purchased from Santa Cruz Biotechnology (Santa Cruz, CA, USA). The antiphospho-p42/p44 ERK (Thr202/Tyr204) was from Cell Signaling (Beverly, MA, USA). The hybond-P polyvinylidene difluoride (PVDF) membrane and enhanced chemiluminescence (ECL) western blotting detection reagent and analysis system were obtained from Amersham (Buckinghamshire, UK). All other chemicals used in this study were of reagent grade.

### 2.2. Human Umbilical Vein Endothelial Cells (HUVECs) Isolation and Culture

Human umbilical cords were obtained from the Hospital of National Taiwan University, and human umbilical vein endothelial cells were isolated by enzymatic digestion as described previously [15]. After 15-min incubation with 0.1% collagenase at 37 ± 0.5°C, umbilical cord vein segments were perfused with 30 mL of medium 199 containing 10 U/mL penicillin and 100 *μ*g/mL streptomycin for the collection of cells. After centrifugation for 8 min at 900 ×g, the cell pellet was resuspended in previous medium supplemented with 20% heat-inactivated fetal bovine serum, 30 *μ*g/mL endothelial cell growth supplement, and 90 *μ*g/mL heparin. Confluent primary cells were detached by trypsin-EDTA (0.05% : 0.02%, v/v), and passages between three and five were used in the experiments. Cultures had typical cobblestone morphology and stained uniformly for human von Willebrand factor (vWF) [16] as assessed by indirect immunofluorescence.

### 2.3. Cell Viability Assay

The viability of HUVECs upon treatment of glucose, brazilin alone, and both combined together was measured by a colorimetric MTT assay. Briefly, HUVECs (2 × 10^5^ cells/well) were seeded on 24-well plates and cultured in DMEM containing 10% FBS for 24 h. HUVECs were treated with glucose at concentrations of (5–150 *μ*M), brazilin (10–100 *μ*M) alone, and pretreated with brazilin (10–100 *μ*M) in glucose (25 and 50 mM) induced cells or an isovolumetric solvent control (0.1% DMSO) for 24 or 48 h. The cell number was measured using a colorimetric assay based on the ability of mitochondria in viable cells to reduce MTT as previously described [17]. The cell number index was calculated as the absorbance of treated cells/control cells × 100%.

### 2.4. Determination of Nitrite Production

Human umbilical vein endothelial cells cultured in 12-well plates were washed twice with Hanks balanced salt solution (HBSS) and then incubated at 37 ± 0.5°C in the same buffer for 30 min with various concentrations of brazilin. Acetylcholine (30 *μ*M) was used as a positive control. Supernatants were collected and then injected into a nitrogen purge chamber containing vanadium (III) chloride in hydrochloric acid at 91 ± 0.5°C. All NO metabolites can be liberated as gaseous NO and reacted with ozone to form activated nitrogen dioxide that is luminescent in red and infrared spectra. The chemiluminescence was detected using a nitric oxide analyzer (NOA280, Sievers Instruments, Boulder, CO, USA) [18]. For calibration, the area under the curve was converted to nanomolar NO using a NaNO_3_ standard curve, and the final data were expressed as pmol/mg protein.

### 2.5. Lipid Peroxidation Assay

Lipid peroxidation was assayed by the thiobarbituric acid (TBA) reaction. The cells were homogenized in ice-cold 1.15% KCl. The samples were used to measure the malondialdehyde (MDA) formed in a peroxidizing lipid system. The amount of thiobarbituric acid reactive substance (TBARS) was determined using a standard curve of 1,1,3,3-tetramethoxypropane.

### 2.6. Measurement of Intracellular ROS

Starved HUVECs (2 × 10^5^ cells/well) were loaded with DCF-DA (20 *μ*M) for 20 min. After treatment with brazilin (100 *μ*M) or a solvent control for 20 min, cells were stimulated with glucose (5.5−75 mM) for 10 min, washed with PBS, and then detached using trypsin. Levels of intracellular ROS were detected by flow cytometry (Beckman Coulter). All experiments were repeated at least four times to ensure reproducibility.

### 2.7. SDS-Polyacrylamide Gel Electrophoresis (PAGE) and Western Blot Analysis

Western blot analyses were performed as previously described [19]. Lysates from each sample were mixed with 6 × sample buffer (0.35 M Tris, 10% w/v SDS, 30% v/v glycerol, 0.6 M DTT, and 0.012% w/v bromophenol blue, pH 6.8) and heated to 95°C for 5 min. Proteins were separated by electrophoresis and transferred onto polyvinylidene difluoride (PVDF) membranes for pERK1/2, p-eNOS, pNF-kB, VCAM-1, and ICAM-1. The membranes were blocked with 5% nonfat milk in TBS-0.1% Tween 20 and sequentially incubated with primary antibodies and HRP-conjugated secondary antibodies, followed by enhanced chemiluminescence (ECL) detection (Amersham Biosciences). Bioprofil Bio-1D light analytical software (Vilber Lourmat, Marue La Vallee, France) was used for the quantitative densitometric analysis. Data of specific protein levels are presented as relative multiples in relation to the control.

### 2.8. Statistical Analyses

The experimental results are expressed as the mean ± SEM and are accompanied by the number of observations. For analysis of the results, a one-way analysis of variance (ANOVA) test was performed using Sigma Stat v3.5 software. When group comparisons showed a significant difference, the Student Newman-Keuls test was used. A *P* value of <0.05 was considered to be statistically significant.

## 3. Results

### 3.1. Effect of Brazilin on HG-Induced HUVECs Cell Viability

**Figure 2:**
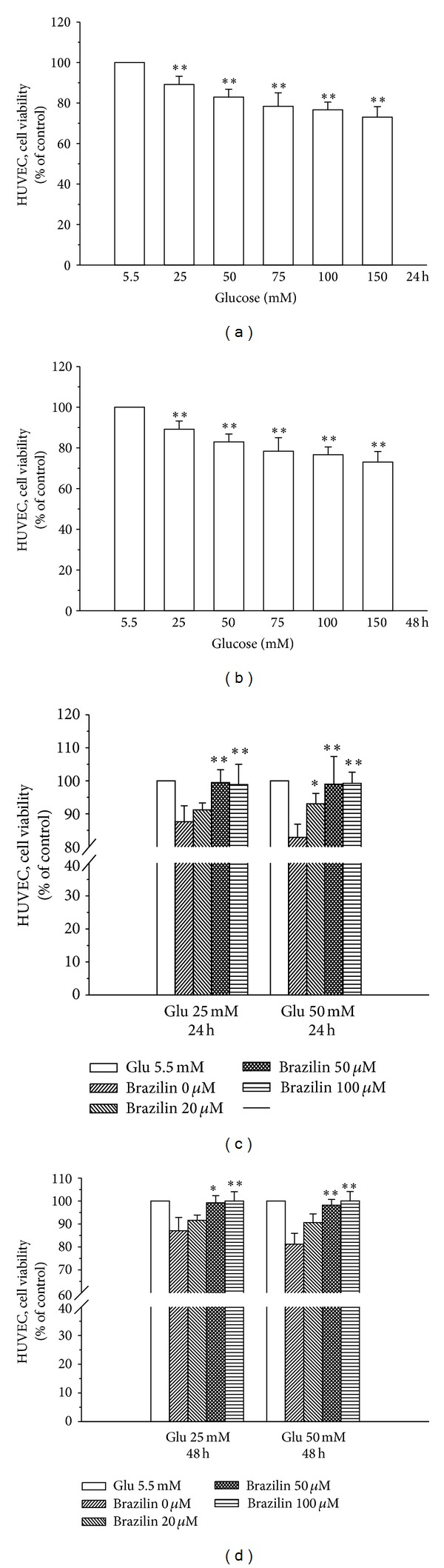
Effects of brazilin on glucose-induced cell viability of human umbilical vein endothelial cells (HUVEC): ((a) and (b)) the viability of HUVECs during treatment with various concentrations (25–150 mM) of glucose for 24 h and 48 h, respectively; ((c) and (d)) the viability of HUVECs during treatment with various concentrations (20–100 *μ*M) of brazilin upon glucose (25 and 50 mM) stimulation for 24 and 48 h, respectively. Data are shown as the mean ± SEM of three independent experiments. *P < 0.05 and ***P < 0.001, compared to the glucose treated group.

Initially, the cytotoxicity of brazilin to HUVECs cells was measured by MTT assay. Cell viability was not significantly altered by brazilin at 10–100 *μ*M for 24 and 48 h with cell viability remaining stable (Figures 1(b) and 1(c)). Interestingly, treatment of cells with glucose (5.5–150 *μ*M) concentration dependently decreased cell viability, whereas cells simultaneously incubated with glucose (25 and 50 mM) and brazilin (0–100 *μ*M) increased cell viability in a concentration-dependent manner (Figures 2(a), 2(b), 2(c), and 2(d)).

### 3.2. Brazilin Inhibits HG-Induced LPO, NO, and ROS in HUVECs

**Figure 3:**
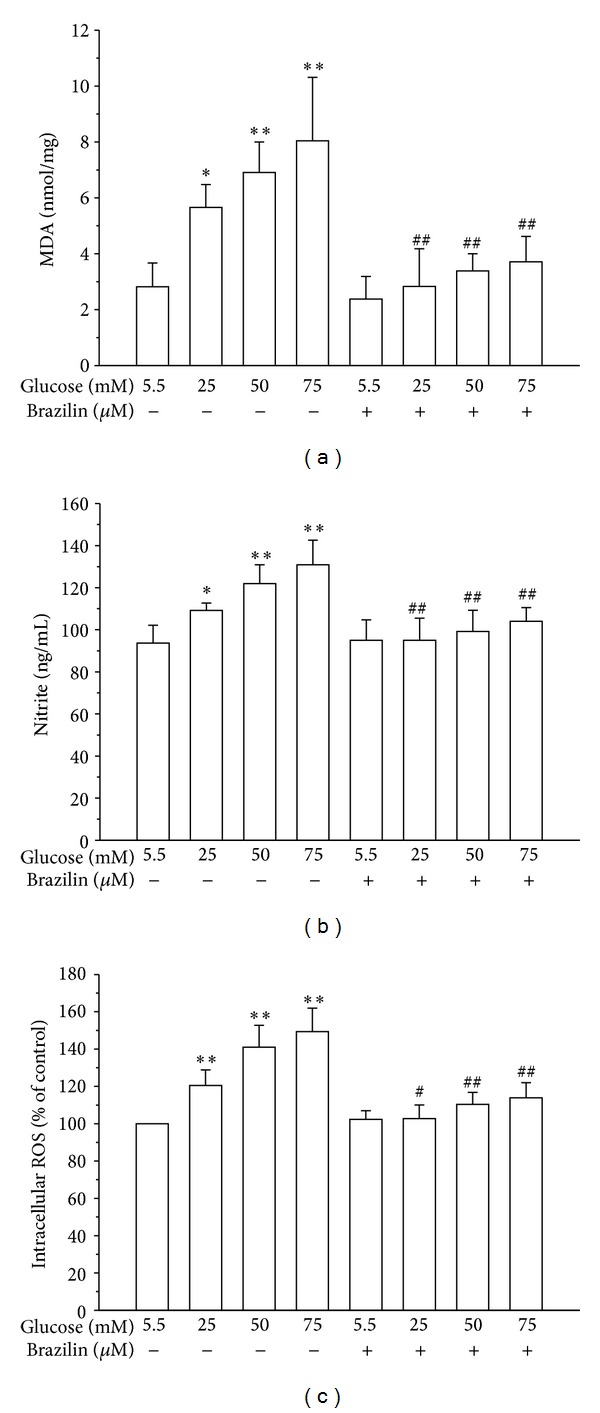
Effects of brazilin on glucose-induced LPO, NO, and ROS production in HUVECs: (a) lipid peroxidation was assayed by measuring the amount of TBARS formation (malondialdehyde, MDA); (b) the nitrite concentration in the culture medium was determined by Griess reagent. (c) ROS production was determined as described in Materials and Methods. Data are shown as the mean ± SEM of three independent experiments. *P < 0.05 and **P < 0.01, compared to the glucose treated group; ^#^ *P* < 0.05 and ^##^ *P* < 0.01, compared to the brazilin treated group.

It is known that damage to cell membranes causes the decreasing of cell viability through peroxidation of membrane lipids. Therefore, we estimated the levels of MDA in the present study. As shown in Figure 3(a), malone-di aldehyde (MDA), a marker of lipid peroxidation, was markedly induced in HUVEC cells by treatment with glucose (5.5–75 mM). However, pretreatment with brazilin (100 *μ*M) significantly reduced glucose-induced lipid peroxidation. Similarly, HUVEC cells treated with glucose (5.5–75 mM) significantly increased nitrite formation in a concentration-dependent manner; however, this increase was markedly suppressed by brazilin at 100 *μ*M (Figure 3(b)). To determine the efficiency of brazilin in inhibiting glucose-induced ROS formation in HUVECs, a cell-permeative ROS-sensitive dye, DCFDA (nonfluorescent in a reduced state but fluorescent upon oxidation by ROS), was used. In this study, glucose (5.5–75 mM) induced ROS formation concentration-dependently as compared to resting (untreated) cells, whereas treatment with brazilin (100 *μ*M) markedly inhibited this formation with the same concentration dependent manner (Figure 3(c)).

### 3.3. Effect of Brazilin on HG-Stimulated Phosphorylations of eNOS and ERK in HUVECs

**Figure 4:**
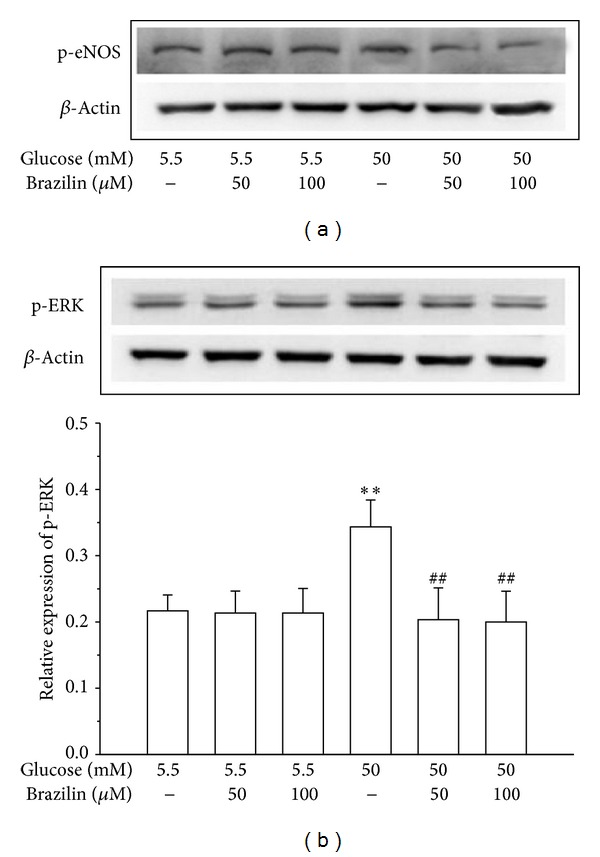
Effects of brazilin on glucose-induced expression of p-eNOS and pERK in HUVECs: HUVECs (2 × 10^5^ cells/well) were pretreated with a solvent control (0.1% DMSO) or brazilin (50 and 100 *μ*M) for 2 h and then treated with glucose (50 mM) for 30 min to detect the phosphorylation of (a) p-eNOS and (b) p-ERK1/2. The *β*-actin was used as an internal control.

To determine whether brazilin affects the activation of p-eNOS and pERK, we analyzed the phosphorylation levels of these proteins. First, HUVECs were pretreated with 50 and 100 *μ*M brazilin for 30 min and then stimulated with 50 mM glucose for 1 h. As shown in Figures 4(a) and 4(b), pretreatment of brazilin concentration-dependently inhibits the HG-induced phosphorylations of p-eNOS and pERK in HUVEC cells.

### 3.4. Effect of Brazilin on HG-Induced NF-*κ*B Activation

**Figure 5:**
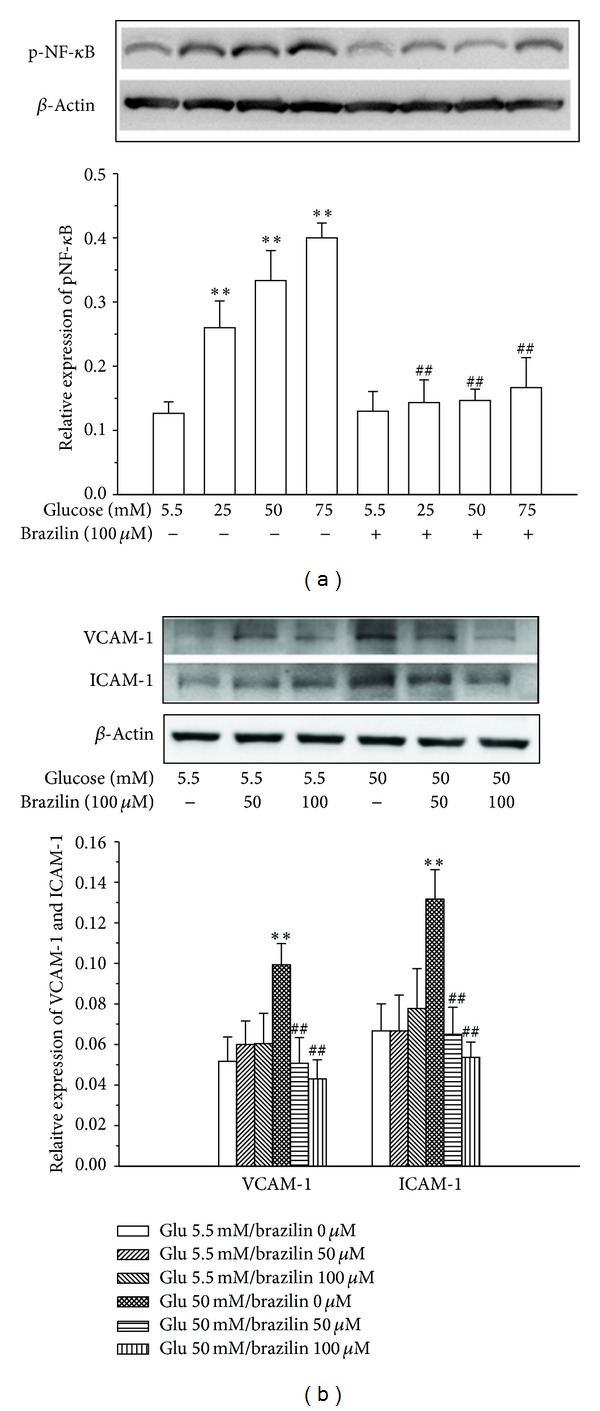
Effects of brazilin on glucose-induced expression of NF-*κ*B and CAMs expression in HUVECs: HUVECs (2 × 10^5^ cells/well) were pretreated with a solvent control (0.1% DMSO) or brazilin (50 and 100 *μ*M) for 2 h and then treated with glucose (50 mM) for 30 min to detect the expression of (a) NF-*κ*B and (b) VCAM-1 and ICAM-1. The *β*-actin was used as an internal control.

ROS has been shown to activate various transcription factors including NF-*κ*B in cultured endothelial cells [20]. In this study, it is proposed that the increased ROS production in HG-induced HUVEC cells may partially cause the activation of NF-*κ*B. Therefore, we measured whether high glucose induced NF-*κ*B activation in HUVECs. Western blotting analysis for NF-*κ*B revealed that there was an increased expression of NF-*κ*B protein in HUVEC treated with high glucose (75 mM). In addition, pretreatment with brazilin (25 and 50 *μ*M) markedly inhibited the high glucose-induced increase of NF-*κ*B expression levels. However, 75 *μ*M of brazilin pretreatment did not affect the NF-*κ*B expression of HG-induced HUVEC (Figure 5(a)).

### 3.5. Effect of Brazilin on HG-Induced Endothelial Adhesion Molecules

To investigate whether glucose induces expression of VCAM-1 and ICAM-1 in HUVECs, we cultured HUVECs at normal glucose (5.5 mM) and high-glucose (50 mM) concentrations for 24 h. Immunoblot analysis showed that stimulation of HUVECs with high concentrations of glucose increased the production of VCAM-1 and ICAM-1 (Figure 5(b)). To further determine whether brazilin can inhibit the expression of endothelial adhesion molecules, we pretreated HUVECs with brazilin at the indicated concentrations (50 and 100 *μ*M) and stimulated the cells with 50 mM glucose for 24 h. As shown in Figure 5(b), pretreatment of brazilin in HUVEC significantly inhibited the HG-induced effect on the ICAM-1 and VCAM-1 levels in a concentration manner.

## 4. Discussion

The present study was undertaken to shed more light on the mechanisms by which brazilin exerts its inhibitory effects on high glucose-induced human umbilical vein endothelial cells. For the first time, we have demonstrated that brazilin inhibited the high glucose-induced vascular inflammation via inhibition of ROS, eNOS, ERK, CAMs, and NF-*κ*B in primary cultured HUVEC. These results indicate that brazilin might be used as a potential candidate for the prevention of hyperglycemia-associated vascular inflammatory processes of endothelial cells. Notably, brazilin showed no cytotoxicity at used concentrations of 0–100 *μ*M in HUVECs for 24 and 48 h stimulations. In line with previous studies [21, 22], the results revealed that incubation of the HUVEC with high glucose (25−150 mM) for 24 and 48 h determined a consistent reduction of cell viability. These results confirmed the detrimental role of hyperglycemia in the HUVEC functionality. However, at the same time, the coincubation with brazilin significantly improved HUVEC viability.

Lipid peroxidation, a process induced by free radicals, leads to oxidative deterioration of polyunsaturated lipids. Under normal physiological conditions, only low levels of lipid peroxides occur in body tissues. The excessive generation of free radicals leads to peroxidative changes that ultimately result in enhanced lipid peroxidation [23]. The endproduct of stable aldehydes reacts with thiobarbituric acid (TBA) to form a thiobarbituric acid-malondialdehyde adduct [24]. In previous studies, it was found that the LPO level was increased in HUVECs when the cells were incubated with glycated protein and iron [25]. In the present study, HG treatment significantly increased lipid peroxidation in HUVECs concentration-dependent manner. Pretreatment of brazilin protected against HG-induced LPO probably because of its ability to inhibit ROS production.

This study further investigated whether brazilin affects oxidative stress via inhibiting ROS and NO production induced by glucose, as several lines of evidence indicated that high glucose induced ROS and NO production. The results revealed that brazilin significantly decreased the glucose-induced ROS and NO production (Figures 3(b) and 3(c)). Previous results of a study suggest that brazilin induces vasorelaxation by the increasing intracellular Ca^2+^ concentration in endothelial cells of blood vessels by activating Ca^2+^-dependent NO synthesis [26]. NO production induced by glucose is reported to be associated with an increase in the expression of eNOS in HUVEC [27], indicating that eNOS is responsible for NO production. There are still arguments about the effect of high glucose on NO production in endothelial cells; a study showed that 30 mM of glucose induced the increase of NO and eNOS at 48 h in HUVEC [28]; however, another study showed that high glucose decreased NO concentration at 7 days in HUVEC cells [29]. In the present study, the results show that pretreatment with brazilin at concentration of 100 *μ*M decreases NO in 25 mM glucose-induced HUVEC, whereas brazilin was not effective in 50 and 75 mM glucose-induced NO. These results may indicate that HUVEC is treated with brazilin as a protective mechanism, and at high glucose (hyperglycemia), it was not cope to reduce NO concentration may due to an enhancement of endothelial inflammation. Endothelial nitric oxide synthase (eNOS) is an important enzyme for the maintenance of cardiovascular function by producing NO. Nevertheless, eNOS can be detached, leading to generation of super-oxide instead of NO under certain pathological circumstances and oxidative stress conditions [30]. In this study, it shows that pretreatment of brazilin predominantly inhibited the phosphorylation of HG-induced endothelial nitric oxide synthase in HUVEC cells.

The production of ROS has been mechanistically associated with inflammatory responses [31, 32]. ROS production and inflammation are potential mediators of diabetes mellitus-associated vascular diseases. Recent study indicates that hyperglycemia activates the generation of free radicals and oxidative stress in various cell types [33]. ROS are considered to be important mediators of several biologic responses, including cell proliferation and extracellular matrix deposition. The formation of oxygen-derived radicals might lead to an activation of NF-*κ*B and ROS-mediated NF-*κ*B activation plays an important role in the pathogenesis of atherosclerosis. Role of ROS and NF-*κ*B in the induction of apoptosis by high glucose has been demonstrated in primary cultured human endothelial cells [34]. In our results, high glucose-induced increment of the production of cellular ROS may suggest that high glucose-induced oxidative stress in HUVEC is important in determining the character of diabetic complication as well as vascular inflammation. In addition, pretreatment with brazilin significantly inhibited the high glucose-induced ROS formation, suggesting a role of protecting vascular inflammation via antioxidant activity.

An interesting finding of this study is that NF-*κ*B might be a target of brazilin against HG-induced cell damage. NF-*κ*B is an ubiquitous transcription factor that manages the expression of genes encoding cell adhesion molecules and some acute phase proteins in health and in various disease states [35]. Thus, advancement of modulatory approaches targeting this transcription factor may provide a novel therapeutic tool for the treatment of various diseases [36]. This transcription factor generally consists of two proteins, a p65 (RelA) subunit and a p50 subunit. In normal condition, NF-*κ*B is bound to its inhibitor protein I-*κ*B, which restricts NF-*κ*B to the cytoplasm, whereas stimulation by cytokines or endotoxin results in the phosphorylation of inhibitor *κ*B, the unbinding of NF-*κ*B from inhibitor *κ*B, and the activation of NF-*κ*B, with its subsequent translocation into the nucleus [37]. This in turn induces the transcription of cell adhesion molecules, chemokines and macrophage migration inhibitory factor, matrix metalloproteinase-1 and 9, and many other genes that regulate transcription, apoptosis, and cell proliferation. Recent reports demonstrated that natural extracts inhibits cell adhesion molecules and monocytes adhesion to endothelial cells via the suppression of NF-*κ*B activation under pathophysiological conditions, including high glucose or cytokines [38]. In the present study, we found that high glucose-induced NF-*κ*B in HUVEC and pretreatment by brazilin blocked the high glucose-induced NF-*κ*B expression in concentration-dependent manner. This result is supported by the previous study that medicinal plant extract such as rhubarb suppressed NF-*κ*B p65 expression in vascular endothelial inflammation process [39]. The finding of this study demonstrated that high glucose-induced NF-*κ*B activation inhibited by brazilin may indicate that brazilin has some inhibitory effect on the NF-*κ*B pathways specific to the high glucose-induced adhesion molecules in HUVEC.

In human umbilical vein endothelial cells, high glucose could make a cellular damage leading to cell apoptosis probably via ERK activation. To make this hypothesis solid, we analysed ERK phosphorylation because the activation of ERK may participate in the defense signaling against oxidative damage in cells [40]. ERK is the signal cascade involved in the protection of oxidative damage and its activation is generally thought to mediate cell survival [41]. In this study, data from Western blot analysis indicate increases in expression of phosphorylated ERK (pERK) in high glucose-treated HUVEC cells, which were blocked by brazilin. Changes in expression of activated ERK closely reflect the cell damage; such results suggesting that high glucose-induced HUVEC cell damage is protected, at least partly, by inhibiting ERK activation.

This study also noticed that high glucose (50 mM) alone increased ICAM-1 and VCAM-1 expression when compared with nontreated control (5.5 mM) and pretreatment with brazilin (Figure 5(b)). These cell adhesion molecules primarily mediated the adhesion of monocytes specifically found in atherosclerosis lesions to the vascular endothelium. Some study showed that high glucose increased only ICAM-1 expression, but not other adhesion molecules [42], whereas other study showed high glucose-induced increased only VCAM-1 expression [43]. These results suggested that high glucose exhibited various effects on CAMs expression. Recently, various phytochemicals have been shown to inhibit the expression of adhesion molecules in endothelial cells. For instance, resveratrol reduced interleukin-6 (IL-6)-induced ICAM-1 expression by interfering with the Rac-mediated pathway through a decrease in the phosphorylation of signal transducer and activator of transcription 3 (STAT3) [44]. Epigallocatechin-3-O-gallate (EGCG) inhibits angiotensin II-induced adhesion molecule expression by inhibiting p38 MAPK and ERK1/2 phosphorylation [27]. Anthocyanins inhibited TNF-*α*-induced ICAM-1 and VCAM-1 expressions via the NF-*κ*B-dependent pathway [45]. Phloretin inhibited the TNF-*α*-stimulated expression of adhesion molecules without activating NF-*κ*B [46]. Grape-seed proanthocyanidin extract inhibited VCAM-1 expression in HUVECs via the NF-*κ*B-independent pathway [47]. Quercetin downregulated ICAM-1 expression in human endothelial cell lines through inhibition of the activator protein-1 (AP-1) and c-Jun NH_2_-terminal kinase (JNK) pathway [48]. Interestingly, we observed that pretreatment with brazilin suppressed HG-induced expression of ICAM-1 and VCAM-1 perhaps via inhibition of the ERK1/2 phosphorylation and NF-*κ*B-dependent pathway. Thus, development of therapeutic drugs for diabetic vascular inflammation targeting CAMs expression may prove useful in the prevention of vascular inflammation.

In conclusion, the most important findings of this study demonstrate for the first time that brazilin was able to eliminate several inflammatory events induced by high concentrations of glucose in HUVEC. In this study, we observed that brazilin reduced CAMs expression in high glucose-induced HUVEC through NF-*κ*B as well as ROS-dependent mechanisms. Therefore, this study suggested that brazilin could be very useful in the treatment of vascular inflammatory process and/or hyperglycemia via inhibition of oxidative stress and NF-*κ*B activation in primary cultured HUVEC.
